# Probing the Structural Dynamics of the Activation Gate of KcsA Using Homo-FRET Measurements

**DOI:** 10.3390/ijms222111954

**Published:** 2021-11-04

**Authors:** Clara Díaz-García, Maria Lourdes Renart, José Antonio Poveda, Ana Marcela Giudici, José M. González-Ros, Manuel Prieto, Ana Coutinho

**Affiliations:** 1 iBB, Institute for Bioengineering and Biosciences, Instituto Superior Técnico, Universidade de Lisboa, 1049-001 Lisboa, Portugal; clara.diaz@tecnico.ulisboa.pt (C.D.-G.); manuel.prieto@tecnico.ulisboa.pt (M.P.); 2 Associate Laboratory i4HB, Institute for Health and Bioeconomy at Instituto Superior Técnico, Universidade de Lisboa, 1049-001 Lisboa, Portugal; 3 Instituto de Investigación, Desarrollo e Innovación en Biotecnología Sanitaria de Elche, Universidad Miguel Hernández, 03202 Elche, Spain; ja.poveda@umh.es (J.A.P.); marcela@umh.es (A.M.G.); gonzalez.ros@umh.es (J.M.G.-R.); 4 Departamento de Química e Bioquímica, Faculdade de Ciências, Universidade de Lisboa, 1749-016 Lisboa, Portugal

**Keywords:** potassium channels, homo-FRET, fluorescence spectroscopy, anisotropy, fluorescent dye, allosteric coupling, ratiometric assay

## Abstract

The allosteric coupling between activation and inactivation processes is a common feature observed in K^+^ channels. Particularly, in the prokaryotic KcsA channel the K^+^ conduction process is controlled by the inner gate, which is activated by acidic pH, and by the selectivity filter (SF) or outer gate, which can adopt non-conductive or conductive states. In a previous study, a single tryptophan mutant channel (W67 KcsA) enabled us to investigate the SF dynamics using time-resolved homo-Förster Resonance Energy Transfer (homo-FRET) measurements. Here, the conformational changes of both gates were simultaneously monitored after labelling the G116C position with tetramethylrhodamine (TMR) within a W67 KcsA background. At a high degree of protein labeling, fluorescence anisotropy measurements showed that the pH-induced KcsA gating elicited a variation in the homo-FRET efficiency among the conjugated TMR dyes (TMR homo-FRET), while the conformation of the SF was simultaneously tracked (W67 homo-FRET). The dependence of the activation p*K*_a_ of the inner gate with the ion occupancy of the SF unequivocally confirmed the allosteric communication between the two gates of KcsA. This simple TMR homo-FRET based ratiometric assay can be easily extended to study the conformational dynamics associated with the gating of other ion channels and their modulation.

## 1. Introduction

Potassium channels are oligomeric proteins that provide an energetically favorable environment for a rapid and highly selective K^+^ conduction across the plasma membrane down its electrochemical gradient [1]. The pore-forming domain is highly conserved and responsible for the permeability and selectivity properties of these proteins [2]. Upon gating, the channels become non-conductive to K^+^ ions through a time-dependent inactivation process, which provides a tight control over the cellular excitability and function [3,4,5]. The presence of different types of modulators, such as the concentration of blocking or permeant cations or different types of lipids, can also modify the conduction, gating, and inactivation properties of the channels [6].

**Figure 1:**
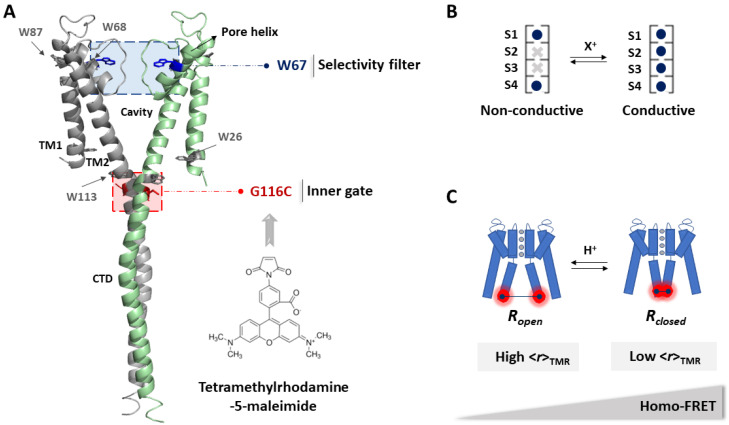
Schematic representation of the KcsA channel and its two gates. (**A**) Full-length structure of KcsA in the closed state (PDB: 3EFF). Two of the four monomers are depicted for clarity. Each monomer consists of two transmembrane helices (TM1 and TM2), a short, tilted pore helix, the selectivity filter (SF), and the C-terminal domain (CTD). KcsA also has five Trp residues per monomer distributed between the intracellular and extracellular mouths of the channel. In this work, W26, 68, 87, and 113 were mutated to phenylalanine, leaving W67 as the sole fluorescent reporter at the pore helix. The G116C mutation was incorporated at the opposite end of the protein to enable its specific conjugation to tetramethylrhodamine-5-maleimide (TMR-M) at this gating-sensitive position. (**B**) The conformation of the SF (outer gate) is modulated by the type and cation concentration X^+^ in solution, adopting either a conductive or a non-conductive conformation. This conformational equilibrium can be tracked by measuring the fluorescence anisotropy of W67, <*r*>_W67_. (**C**) The conformational shift of the inner gate of KcsA between an open and a closed conformation is modulated by the H^+^ concentration in solution. The opening of this gate can be monitored by an increase in the TMR fluorescence anisotropy, <*r*>_TMR_, due to a decrease in the efficiency of the intramolecular homo-FRET process among the multiply conjugated dyes to a single channel.

The KcsA channel from *S. lividans* is a homotetrameric membrane protein considered as a prototype for the study of K^+^ channels function and modulation, not only due to its simple structure but also to its homology to its eukaryotic counterparts [7]. Each channel subunit consists of two α-helical transmembrane segments (TM1 and TM2) and a N- and C-terminal cytoplasmic ends per monomer (Figure 1). The four C-terminal ends of the TM2 sections arrange as a helical bundle with a conformation that is sensitive to pH, acting as an intracellular gate (inner or activation gate) [8,9]. The conduction pathway is delimited by the continuum of an aqueous cavity and the selectivity filter (SF) with the highly conserved signature sequence TVGYG [7]. The ability of this later domain to adopt several non-conductive and conductive conformations defines it as the second gate (extracellular outer or inactivation gate) [2,10,11].

The conformational plasticity of the SF can be modulated by the type and concentration of cations within the pore [11,12], the inactivation triad (a network of interactions with residues located at the short, tilted pore helix, including E71, D80, and W67 [13]), and by the lipid composition of the membrane [6,14]. Furthermore, several studies have suggested that both gates are allosterically coupled through the connection of the opening of the intracellular gate to the SF conformation by concerted structural rearrangements of several key amino acids from the aqueous central cavity [13,15]. After gating by a pH drop, the intracellular gate of KcsA widens up to the hinge point, G99. Even though the upper portion of the pore is not widely affected, the side chains of F103, I100, and M96 translate the motion to the SF, which enters into a non-conductive form, the inactivated state [16]. Likewise, it has also been reported that changes in the SF conformation related to its K^+^ occupancy can also modify the open-closed equilibrium of the inner gate [17,18,19]. As in KcsA, it is presumed that most other K^+^ channels are controlled through two similar allosterically coupled gates at both ends of the permeation pathway, even though the molecular details of such regulatory mechanisms are still unclear [2,5,20].

We have been studying the conformational dynamics of KcsA through the application of advanced homo-FRET methodologies [12,21]. FRET is a non-radiative distance-dependent photophysical process where energy is transferred from an excited fluorescent donor (D) to a suitable ground-state acceptor (A) via a long-range dipole-dipole coupling mechanism [22]. The Förster theory states that the energy transfer rate constant is inversely related to the sixth power of the distance separating the two interacting molecules, and so FRET measurements have been widely used as a spectroscopic molecular ruler [23]. FRET can not only happen between chemically distinct donors and acceptors (hetero-FRET) but also occurs between identical molecules (homo-FRET) that typically display a small Stokes shift. In this case, the fluorescence properties (quantum yield and fluorescence lifetime) of the donating and accepting molecules are indistinguishable, and these traditional readouts of a hetero-FRET experiment cannot be used. However, each energy transfer step within a cluster of identical fluorophores leads to a FRET-induced angular displacement of the emitted fluorescence that can be detected through polarized fluorescence measurements [24]. Homo-FRET or energy migration within a population of identical fluorophores can therefore be tracked by measuring the fluorescence anisotropy of the sample which provides information about their ensemble averaged separation distance [12,25,26]. Homo-FRET approaches have been increasingly used to study both intramolecular and intermolecular interactions, not only in solution but also in live cells through the implementation of fluorescence anisotropy imaging microscopy (FAIM) [27,28]. The pioneering work of Johansson and collaborators set the stage for determining distances by fluorescence energy homotransfer [25]. This technique has also been used to detect conformational changes [29] and protein oligomerization [30] in solution. More recently, genetically expressed biosensors based on intramolecular homo-FRET have been developed [31,32,33] and this methodology has also been used for detecting protein clustering and self-assembly in live cells [26,34].

In a previous study, we developed a single Trp mutant of KcsA by silencing the fluorescent signal from four out of the five Trp residues per monomer (W26, 68, 87, 113F KcsA mutant channel, so-called W67 KcsA from now on) (Figure 1A) [12]. Polarized steady-state and time-resolved fluorescence measurements were then used to study tryptophan-tryptophan homo-FRET in the detergent-solubilized W67 KcsA mutant channel to characterize the interplay between the pore helix and the SF conformation. We found that the tryptophan 67 residues were able to sense the concerted structural changes related to the transitions between the different biological intermediates (blocked, conductive, or inactivated) (Figure 1B) [12,21]. To further extend these studies to the activation gate, a new homo-FRET based sensor capable of detecting the structural rearrangements of the inner gate after the pH-induced activation was engineered in W67 KcsA (Figure 1C). To do so, the native glycine at position 116 was mutated to a cysteine residue within the W67 KcsA sequence. This position is located at the end of the TM2 helix and has already been shown to be sensitive to the opening and closing of the inner gate in several EPR studies [35,36,37]. Due to the absence of native Cys residues in KcsA, cysteine 116 allows for its subsequent specific derivatization with a fluorescent probe following the maleimide chemistry. The selected fluorescent dye for labeling G116C W67 KcsA was tetrametylrhodamine-5-maleimide (TMR-M) (Figure 1A). In general terms, this fluorescent dye has a high photostability, relative pH insensitivity, red-orange fluorescence, and its thiol conjugates have strong absorptivity and high fluorescence quantum yields [38]. TMR-M was first used by Francisco Bezanilla and collaborators to detect the pH-induced opening of the inner gate of this channel using fluorescence lifetime measurements in the frequency domain [39]. A similar approach was applied in voltage-clamp fluorometry studies, wherein the TMR is attached to cysteine residues in the voltage-sensor domains of ion channels, thus generating changes in the fluorescence intensity of the dye that are responsive to structural rearrangements associated with channel gating [40,41].

**Figure 2:**
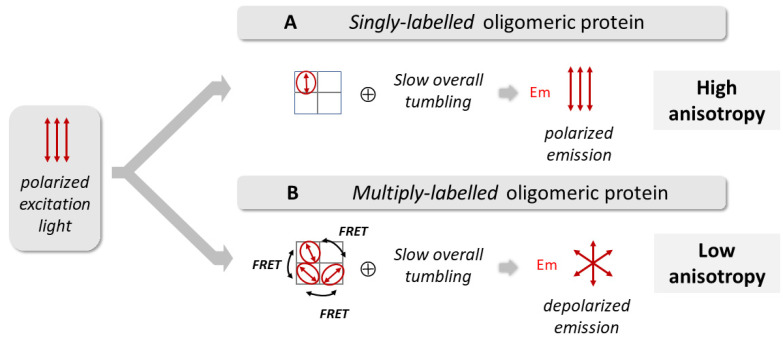
The degree of protein labelling strongly influences the steady-state fluorescence anisotropy of a homo-oligomeric protein due to the possible occurrence of an intramolecular homo-FRET process in the multiply labeled species. The experimental readout used to detect the occurrence of FRET among identical fluorophores with a small Stoke shift (homo-FRET) is the steady-state fluorescence anisotropy of the sample, <*r*>. This requires the performance of polarized fluorescence measurements whereupon the sample is excited with vertically polarized light and the parallel and perpendicular components of the emitted fluorescence are used to compute <*r*> (Equation (2)). (**A**) At a low degree of protein labelling, the predominant labeled species in solution is the singly labeled homo-oligomeric protein. In this case, the main factor responsible for the depolarization of the emitted fluorescence is the slow overall tumbling of the protein in solution over time, and therefore the fluorescence anisotropy, <*r*>, is very high. (**B**) However, at a high degree of protein labelling, a heterogeneous population of multiply labeled species is present in solution. When the multiple fluorophores attached to a single protein are in close proximity, successive intramolecular FRET steps (energy migration) among the conjugated dyes are highly probable. This leads to a strong FRET-induced angular displacement (depolarization) of the emitted fluorescence, which results in a pronounced decrease of the sample fluorescence anisotropy, <*r*>.

The simultaneous presence of two independent fluorescent reporters, one at each gate of the channel (W67 at the SF and TMR at the inner gate, respectively (Figure 1A)) allows to investigate the allosteric crosstalk between the inner gate and the SF during KcsA gating. W67 is a genetically encoded fluorescent reporter and hence is present in all G116C W67 KcsA subunits. However, the random labeling of an oligomeric protein with one labeling site per subunit results in a heterogeneous population with distinct subpopulations of fluorescently labeled channels with a variable number of covalently linked probes (Figure S1) [42]. Therefore, the homo-FRET studies of the tetrameric KcsA channel conjugated to the extrinsic fluorescent dye first required studying the influence of the degree of protein labeling on this process. An under labeled sample, predominantly consisting of singly labeled proteins, allows for the characterization of the rotational dynamics of the conjugated dye/protein and to study the influence of pH on its fluorescence properties (Figure 2A). On the other hand, samples prepared with a higher degree of labeling, which are enriched in multiply labeled protein species, give information about the extent of the homo-FRET process among the fluorescently labeled subunits within each tetrameric channel (Figure 2B) which, in turn, is sensitive to the conformational transition between an open/closed state of the inner activation gate (Figure 1C).

By combining steady-state and time-resolved fluorescence anisotropy measurements of the TMR-labeled G116C W67 KcsA channels and their ability to detect homo-FRET, we were able to monitor the protonation-sensitive conformational switch of the inner gate and its allosteric connection to the SF (outer gate). This study further provides a proof-of-concept that a simple homo-FRET based assay, which relies on using steady-state anisotropy measurements (a ratiometric parameter), can be used to monitor the conformational dynamics associated with the gating of ion channels.

## 2. Results

### 2.1. The G116C W67 KcsA Mutant Behaves as a Wild-Type Channel

**Figure 3:**
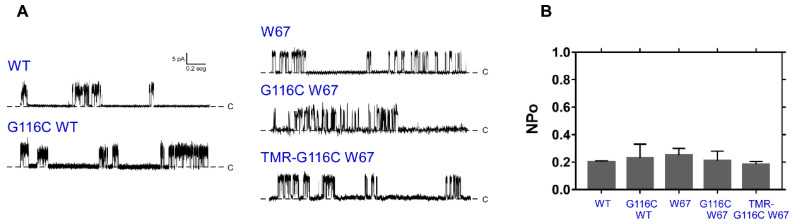
The mutation G116C and its conjugation to TMR does not impact on the functional properties of the wild-type (WT) and W67 KcsA channels. (**A**) Inside-out patch-clamp recordings in continuous mode at +150 mV of WT [12], G116C WT, W67 [12], G116C W67 KcsA, and TMR-labeled G116C W67 KcsA (dye-to-protein molar ratio, D:P = 0.45) channels reconstituted in asolectin liposomes (10 mM HEPES, 100 mM KCl at pH 7.0). The baseline (dashed lines) indicates the closed channel state (closed, C). Channel openings appear as upward deflections over the closed state line. The WT and G116C WT protein showed the expected behavior previously reported in the literature [43,44]. (**B**) The open channel probability, NP_o_ is presented as the average ± SD of at least three independent experiments.

The functional and structural properties of the new mutant channel, G116C W26, 68, 87, 113F (G116C W67 from hereafter) KcsA, were first characterized and compared to the W67 variant of KcsA [12]. In parallel, the properties of WT and G116C KcsA mutant were also evaluated as additional controls. The results obtained by a combination of patch-clamp recordings (Figure 3), thermal stability assays and SDS-PAGE (see Supplementary Information section, Figure S2) ensured that the G116C W67 and WT KcsA mutants behave as their W67 and WT counterparts, respectively. TMR conjugation to cysteine 116 was also found to not significantly perturb the functionality and tetrameric structure of the fluorescently labeled channels.

### 2.2. The Spectroscopic Properties of TMR Conjugated to G116C W67 KcsA Protein at a High Degree of Labeling Are Responsive to Channel Gating Induced by a pH Drop

The labelling efficiency of the G116C W67 KcsA mutant (final dye-to-protein molar ratio, D:P) was calculated by independently quantifying the concentrations of conjugated TMR dye and protein (see the Experimental Procedures section for more details). The final labeling ratio could be modulated by varying the dye:protein molar ratio used during the chemical reaction of the cysteine residues with the TMR-M dye. We first sought to characterize the influence of the degree of protein labeling on the spectroscopic properties of the TMR dye covalently linked to G116C W67 KcsA at distinct titration points within the pH range of 3.5 to 7.0. The measurements were conducted in the presence of a saturating concentration of KCl as in this condition the inner gate and the SF transit respectively from a closed and conductive state at pH 7.0, to an open inactivated state at pH 4.0 [10,45]. The overall yield of the labeling reaction strongly impacts the distribution of fluorescently labeled channels with a variable number of conjugated fluorescent probes. Assuming that the G116C variant of W67 KcsA is randomly labeled with TMR-M [46], the fraction of *k* fluorescently labeled monomers per tetrameric channel, *P*(*k*) can be calculated as a function of its degree of labeling using a binomial distribution. Upon increasing the final dye-to-protein molar ratio, D:P, the samples progressively shift from consisting of predominantly unlabeled and single labeled species to a heterogeneous population of multiply labeled species (Figure S1). For example, at a low degree of labeling (D:P = 0.12), the normalized percentages of single and double labeled proteins are 82% and 17%, respectively. When the protein labeling yield is raised to D:P = 0.45, the fluorescently labeled tetramers become broadly distributed among single (33%), double (40%), triple (22%), and fully labeled channels (5%), respectively.

**Figure 4:**
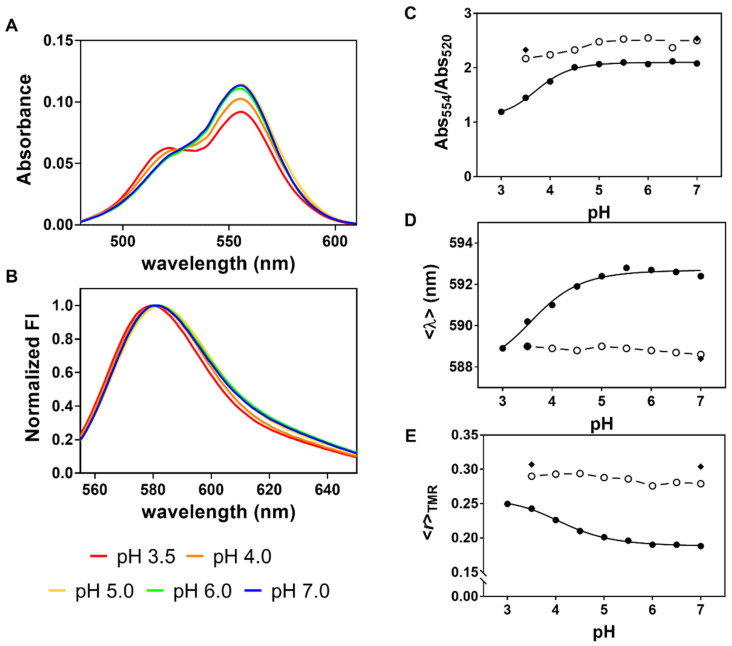
Influence of pH on the spectroscopic properties of the TMR reporter conjugated to the G116C W67 KcsA protein at variable D:P ratios. The samples were prepared with a D:P = 0.05 (◆), 0.12 (○), or 0.45 (●). (**A**) Absorbance and (**B**) normalized emission spectra (λ_ex_ = 490 nm) of the TMR conjugated protein (D:P = 0.45) at pH 3.5, 4.0, 5.0, 6.0, and 7.0. The absorption spectra are characterized by the progressive appearance of a shoulder at ~520 nm corresponding to H-type dimer formation at acidic pHs. The emission spectra present a small red-shift at neutral pHs which is putatively assigned to residual oblique J-type dimer formation. (**C**) The extent of H-dimer and (**D**) putative oblique J-dimer formation for the labeled proteins were monitored by the change in the Abs_554_/Abs_520_ ratio and the spectral center-of-mass, Δ<λ>, with pH, respectively. (**E**) Variation of the steady-state fluorescence anisotropy of the TMR reporter present in TMR-labeled G116C W67 KcsA, <*r*>_TMR_, with pH at different degrees of labeling. The measuring conditions were λ_ex_ = 490 nm; λ_em_ = 580 nm. Error bars are smaller than the symbols in most cases. The solid lines (D:P 0.45) correspond to the empirical fits of a Hill equation to the sigmoidal behavior of each data set with pH, giving a (**C**) p*K*_a_(K^+^) = 3.8 ± 0.1, (**D**) p*K*_a_(K^+^) = 3.7 ± 0.2, and (**E**) p*K*_a_(K^+^) = 4.2 ± 0.1, respectively. The dashed lines are just a guide to the eye. The samples contained 5 mM DDM and 200 mM KCl (*T* = 25 °C).

Both the absorption and emission spectra of the TMR-conjugated channels obtained with a high fractional labeling (D:P 0.45) varied with pH, in contrast to the spectroscopic data obtained with a much lower D:P of 0.05 or 0.12, which were found to be essentially pH-independent (Figure 4). At pH 7.0, the absorption spectrum of TMR conjugated G116C W67 KcsA (D:P 0.45) presented a peak at 554 nm and a small shoulder at 520 nm, that became increasingly more important as the pH of the solutions decreased (Figure 4A). These spectral alterations were not concomitantly detected in the excitation spectra of the fluorescently labeled protein (data not shown) and can be assigned to H-type dimer formation by the conjugated TMR dyes [47,48]. These are non-fluorescent (dark) complexes, which were clearly favored here by the opening of the inner gate of KcsA at acidic pH. In fact, rhodamine dyes have an intrinsic high propensity to aggregate [49,50] and the maximum peaks detected in their absorbance spectra (λ = 520 and 554 nm) are in agreement with the ones reported for a pure H-dimer in a water:ethanol mixture (λ = 525 and 553 nm) [51]. The sigmoidal dependence of the extent of H-dimer formation with pH, evaluated from the absorbance ratio between the two predominant peaks, was well described by a Hill equation with an apparent activation p*K*_a_(K^+^) = 3.8 ± 0.1 (Figure 4C).

In parallel, the collected emission spectra of TMR covalently bound to G116C W67 (D:P 0.45) were slightly red shifted upon increasing the pH from 3.5 to 7.0 (Δ<λ>~2–3 nm), (Figure 4B,D). This shift is caused by a small broadening of the emission band of the conjugated dye, most probably due to residual oblique J-type dimer formation, as previously described for rhodamine [52,53] and BODIPY dyes [54,55]. Interestingly, the formation of these putative fluorescent oblique J-dimers that emit at long wavelengths is now favored at neutral pH when the cytoplasmic gate of the channel is closed, i.e., when the CTDs of the subunits are extended and highly sterically constrained due to their association [56,57]. An apparent p*K*_a_(K^+^) of 3.7 ± 0.2 was obtained from empirically fitting a Hill model to the sigmoidal variation of <λ> with pH (D:P 0.45, Figure 4D). In contrast, the emission spectra of under labeled TMR-labeled G116C W67 KcsA samples presented a constant <λ>~589 nm (D:P 0.05 and 0.12, Figure 4D), in agreement with the expectation that the predominant fluorescently labeled species in solution are the singly labeled proteins. This result effectively rules out that pH is the factor responsible for the spectral alterations detected at a higher D:P.

Lastly, we run several parallel controls using the free detergent-solubilized TMR-M dye in the same buffers to ensure that the presence of trace amounts of free dye is not responsible for the above-described behavior (Figure S3).

### 2.3. The Fluorescence Anisotropy of Multiply TMR-Labeled G116C W67 KcsA Is a Sensitive Reporter of pH Gating Due to an Efficient Intramolecular Homo-FRET Process

To gain further information related to the structural dynamics of the pH-induced channel gating, the steady-state fluorescence anisotropy of the TMR reporter, <*r*>_TMR_ (inner gate) was also measured for the TMR labeled samples described above (Figure 4E). First, a very high <*r*>_TMR_~0.31 was obtained at both pH 3.5 and 7.0 when the singly labeled protein is predominant in solution (D:P 0.05). However, when the degree of protein labeling was increased to 0.12, and particularly to 0.45, much lower <*r*>_TMR_ were measured for the TMR-conjugated channels along the entire pH curve (Figure 4E) This behavior is a strong indicator of an efficient intramolecular homo-FRET process among the conjugated dyes in each of the multiply labeled protein species (Figure 2 and Figure S1). Certainly, the small Stokes shift of the TMR-conjugated dye results in a large Förster radius of *R*_0_~44 Å due to the high overlap between its absorption and emission spectra, making this a highly efficient photophysical process among the fluorophores at a close distance. The pronounced sigmoidal decrease of <*r*>_TMR_ with pH at a high D:P can thus be explained by the pH-switchable conformation of the cytoplasmic KcsA gate: at neutral pH, the inner gate is closed and so energy migration along the multiply attached TMR dyes to a single channel protein is highly likely due to their close proximity which, in turn, leads to a strong depolarization of the emitted fluorescence (low <*r*>_TMR_). On the other hand, the untangling of the inner helical bundle at acidic pH causes an increase in the intersubunit distances [58,59], and therefore results in less homo-FRET among the fluorescently-labeled subunits due to the strong dependence of the energy transfer rate constant with 1/*R*^6^ (higher <*r*>_TMR_) (Figure 1C). A p*K*_a_(K^+^) = 4.2 ± 0.1 was obtained from the empirical fit of <*r*>_TMR_ versus pH with the Hill equation (D:P = 0.45, Figure 4E). Several experiments conducted with independently prepared TMR-labeled G116C W67 KcsA batches confirmed that the activation p*K*_a_ was independent of the degree of protein labeling (D:P~0.40–0.60).

To further corroborate our conclusions, time-resolved fluorescence and anisotropy measurements were also performed to complement the steady-state characterization of the samples. We found that the mean fluorescence lifetime of the TMR-labeled channels (<τ>~3.4 ns) was essentially independent of the experimental conditions used, that is, the degree of protein labeling (D:P) and pH (Figure S4). This reinforces the conclusion that intramolecular homo-FRET among the multiple fluorescently labeled subunits of each tetramer is the major factor responsible for the large changes detected in <*r*>_TMR_ with the final D:P of the sample within the 3.5 to 7.0 pH range. In fact, the invariance of <τ> along the pH titration curve indicates that the data is not significantly influenced by a possible hetero-FRET component from TMR monomers to H-type dimers at low pH. On the other hand, the use of KcsA channels with a single Trp residue per subunit might explain why the large conformational changes undergone by the inner gate were not accompanied by any quenching of the TMR fluorophore, as it is often found in other systems (e.g., [60], including WT KcsA [39]). Both the W26 and W113 residues positioned near the inner gate have been mutated to Phe in G116C W67 KcsA, and therefore its single W67 residue, which is positioned behind the SF, is very far away from the TMR conjugated dyes (≈36 Å, G116-αC-W67-αC distance) (Figure 1).

**Figure 5:**
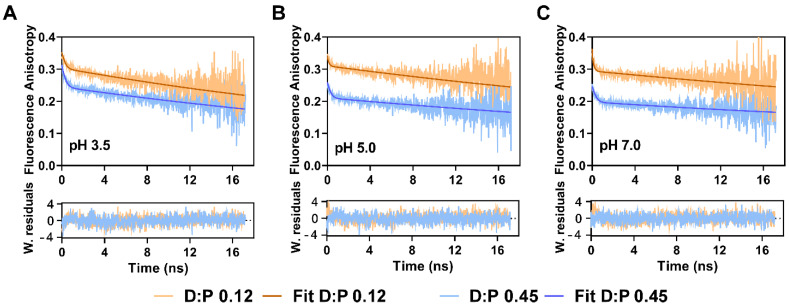
Representative fluorescence anisotropy decays of the TMR conjugated to G116C W67 KcsA protein at (**A**) pH 3.5, (**B**) pH 5.0, and (**C**) pH 7.0. The labeling ratio, D:P, of the samples was 0.12 (orange data) and 0.45 (blue data). The solid lines are the best fits of a double exponential function to the experimental data and the fitted parameters are summarized in Table 1. Bottom panels: the weighted residuals (W. residuals) are randomly distributed around zero showing the goodness of the fits. The measuring conditions were λ_ex_ = 488 nm λ_em_ = 575 nm. The buffers contained 5 mM DDM and 200 mM KCl (*T* = 25 °C).

Illustrative anisotropy decay curves obtained at different pHs for the TMR conjugated G116C variant of W67 KcsA with a D:P 0.12 and 0.45 are also presented in Figure 5. All anisotropy decays were well described by considering two rotational correlation times, a short depolarizing component, φ_1_, associated with the fast local motion of the TMR dye covalently bound to the protein backbone, and a very long one, φ_2_, assigned to the slow overall tumbling of the detergent-solubilized protein in solution. To minimize the error in the fitting procedure, a global analysis of the anisotropy decay curves obtained at each pH/ionic condition was performed by linking φ_2_ between the decays measured for the TMR-conjugated protein with a fractional labeling of 0.12 and 0.45 [22]. In fact, it is well known that the lifetime, τ, of the fluorescent probe circumscribes the measured rotational correlation times and that, as rule-of-thumb, the approximate range of recoverable correlation times with high precision lies within 0.1 τ < φ < 10 τ [61]. The anisotropy decay curves displayed three important features: first, the φ_2_ values were expectedly much longer than the ones obtained for the DDM micelles using the free TMR-M dye (φ_2_~28 ns, Figure S5) since now the fluorophore is covalently linked to the protein; in addition, there was a clear trend for an increase of φ_2_ with pH (Table 1). Notably, these time-resolved anisotropy measurements were sensitive enough to detect a transition from a mostly dissociated, loose CTD at an acidic pH to a more rigid and fully extended CTD at neutral pH in agreement with the literature [56,57]. Secondly, for a D:P 0.12 and under all experimental conditions tested, the time-zero anisotropy, *r*(0) = β_1_ + β_2_ = 0.38 ± 0.01 was very close to the fundamental anisotropy of the TMR dye (*r*_o_ ≈ 0.4 [62]), revealing that the anisotropy decays of the predominantly single-labeled protein fully captured the depolarization of the fluorescence emitted by the conjugated dye over time. The small amplitude, β_1_, associated with the initial fast depolarization of the emitted fluorescence is justified by the absence of a linker in TMR-M (Figure 1). Finally, for the samples prepared with a D:P 0.45, the initial depolarization of the emitted fluorescence was so fast that was beyond the time resolution of our equipment (*r*(0) = β_1_ + β_2_ << 0.40) (Table 1). Indeed, this result is the experimental signature for the occurrence of a very fast homo-FRET process among several fluorescently-labeled subunits within a protein oligomer [34,63]. This effect was also more pronounced at neutral compared to acidic pH values, i.e., when the inner helix bundle crossing stabilizes the closed conformation of the channel (Figure 1C), thus confirming that the low <*r*>_TMR_ values measured under this experimental condition are due to a highly efficient homo-FRET process. It should be further noted that the determination of inter-subunit distances through the application of advanced homo-FRET formalisms, as it was previously done for W67 KcsA [12,21], was not implemented here since it would be necessary to take into account the complex photophysics described above for this probe.

### 2.4. The Ion Occupancy of the SF Modulates the Allosteric Coupling of the Gates

To verify the allosteric crosstalk between the two gates of KcsA, TMR-labeled G116C W67 KcsA samples (D:P ≥ 0.4) were also studied under different ionic conditions. We included measurements at 200 mM K^+^ or Rb^+^ as representatives of a conductive SF at neutral pH, and 200 mM Na^+^, where the SF is expected to be in a collapsed form [10,11]. In this way, the activation p*K*_a_ the inner gate of KcsA, retrieved from the variation of <*r*>_TMR_ with pH, can be correlated with the conformation adopted by the SF, as reported by <*r*>_W67_ [12,21].

**Figure 6:**
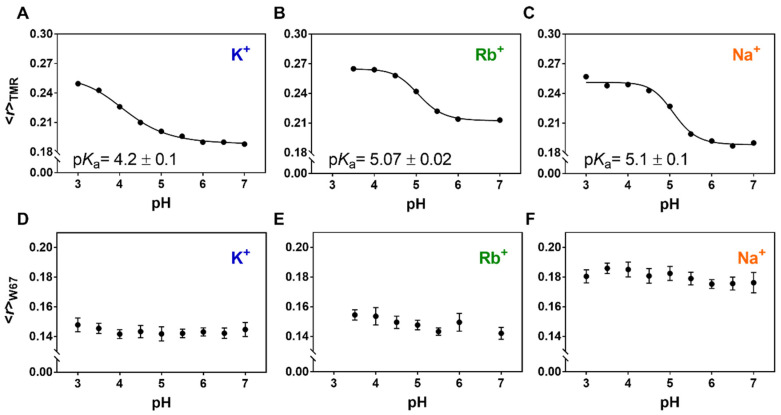
Influence of SF ion occupancy on the activation p*K*_a_ of KcsA. Variation with pH of the steady-state fluorescence anisotropy of the conjugated TMR, <*r*>_TMR_ (**A**–**C**), and W67, <*r*>_W67_ (**D**–**F**) in TMR-labeled G116C W67 KcsA. The experiments were conducted in the presence of 200 mM (**A**,**D**) KCl (D:P 0.45), (**B**,**E**) RbCl (D:P 0.38), and (**C**,**F**) NaCl (D:P 0.45). The measuring conditions were <*r*>_TMR_: λ_ex_ = 490 nm λ_em_ = 580 nm and <*r*>_W67_: λ_ex_ = 300 nm λ_em_ = 340 nm. The empirical fits of a Hill equation to the sigmoidal behavior of <*r*>_TMR_ vs. pH data (solid lines) show a (**A**) p*K*_a_(K^+^) = 4.2 ± 0.1, (**B**) p*K*_a_(Rb^+^) = 5.07 ± 0.02 and (**C**) p*K*_a_ (Na^+^) = 5.10 ± 0.07, respectively. The buffers also contained 5 mM DDM (*T* = 25 °C).

For each system studied, <*r*>_TMR_ displayed the expected sigmoidal dependence with pH and an apparent activation p*K*_a_(Rb^+^) = 5.07 ± 0.02 (Figure 6B) and p*K*_a_(Na^+^) = 5.10 ± 0.07 (Figure 6C) were obtained from fitting a Hill equation to the experimental data. Both of these values are higher than the one obtained in KCl (p*K*_a_(K^+^) = 4.2 ± 0.1, Figure 6A), evidencing a destabilization of the inner gate in the presence of both RbCl and NaCl. The spectroscopic behavior of these samples was similar to the one described for KCl: the absorbance spectra of the TMR-labeled G116C W67 KcsA revealed again the formation of non-fluorescent H-type dimers by the covalently attached dyes at acidic pH (Figure S6A), whereas a residual presence of the putative oblique J-type dimers was inferred from the changes of their emission spectra near neutrality (Figure S6B). In addition, the mean fluorescence lifetime of these samples was also found to be essentially independent of the sample pH (Figure S7C).

Parallel fluorescence anisotropy measurements from the outer gate reporter W67, <*r*>_W67_, confirmed that the SF adopted the expected conformation for each ionic condition tested, even with the TMR dyes covalently linked near the inner gate: characteristically lower values were obtained in the presence of a saturating concentration of K^+^ and Rb^+^ (<*r*>_W67_(K^+^) = 0.144 ± 0.002 (Figure 6D) and <*r*>_W67_(Rb^+^) = 0.149 ± 0.005 (Figure 6E), respectively) due to an highly efficient homo-FRET process among the W67 residues in a conductive SF conformation [12,21], compared to <*r*>_W67_ = 0.180 ± 0.004 in the presence of 200 mM Na^+^ (Figure 6F), when the SF displays a collapsed conformation. Therefore, the lower activation p*K*_a_ obtained for the KcsA inner gate in 200 mM KCl compared to RbCl and NaCl seems to be conditioned not only by the conformational state of the SF (conductive vs. collapsed) but also by its average ion occupancy. Indeed, although both permeant ions K^+^ and Rb^+^ induce a conductive SF at neutral pH, the SF displays a diminished ion occupancy in the second case: the crystal structures of KcsA solved in the presence of Rb^+^ ions characteristically miss an ion at the second K^+^ binding site [10,64] in contrast to the SF in the presence of K^+^, which displays an average occupancy of 2 ions, either in the S1–S3 or the S2–S4 positions [10]. Hence, the closed conformation of the inner gate is more stable only when the SF is in the presence of high K^+^ amounts (i.e., when there is an equal occupancy of the four binding sites). On the other hand, a lower average ion occupancy of the SF (200 mM Rb^+^ or Na^+^) destabilizes the outer gate which, in turn, results in a higher activation p*K*_a_, thus providing definitive evidence for the existence of an allosteric coupling between the two gates of the KcsA channel as previously reported [18].

It should also be stressed that the anisotropy data for W67 is not affected by FRET between this probe and the TMR dye conjugated to the protein due to the small Förster radius for Trp to TMR and by the large separation distance between the two fluorophores (Figure 1A).

### 2.5. The Truncation of the C-Terminal Domain of KcsA Modestly Influences the Sensitivity of the Inner Gate to Protons

**Figure 7:**
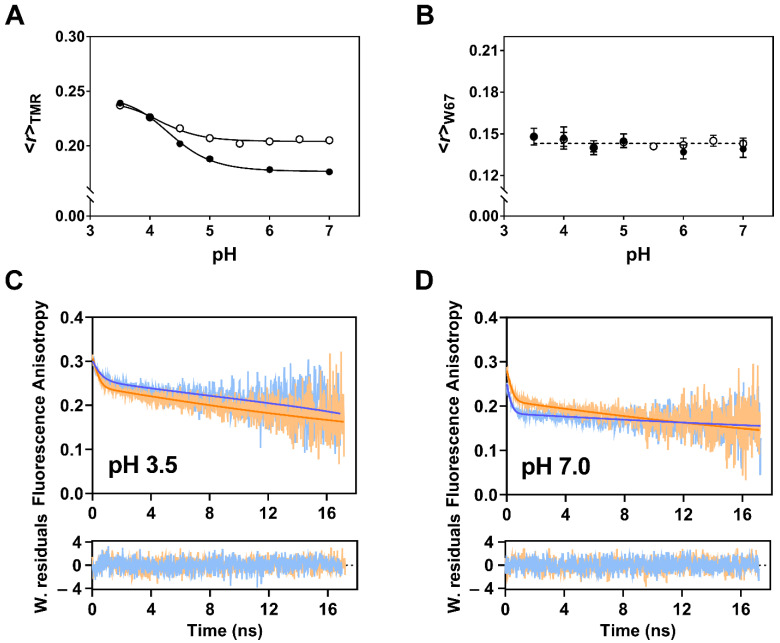
Impact of C-terminal truncation on the conformational dynamics of the KcsA inner gate. (**A**,**B**) The steady-state fluorescence anisotropy of the (**A**) conjugated TMR, <*r*>_TMR_, and (**B**) W67, <*r*>_W67_, reporters were measured for the full-length (FL, ●) and proteolytically cleaved (Δ125, ○) TMR-labeled G116C W67 KcsA (D:P = 0.60). The measuring conditions were as in Figure 6. (**A**) The fits of a Hill equation to the sigmoidal data (solid lines) yielded a p*K*_a_(FL)= 4.34 ± 0.04 and p*K*_a_(Δ125) = 4.3 ± 0.1. (**B**) The dashed line corresponds to an average <*r*>_W67_ = 0.142 ± 0.003 (*n*= 14). (**C**,**D**) Anisotropy decays of TMR conjugated to G116C W67 KcsA (D:P 0.60) performed at (**C**) pH 3.5 and (**D**) 7.0 for the full-length (FL, blue data) and proteolytically cleaved (Δ125, orange data) protein (λ_ex_ = 488 nm λ_em_ = 575 nm). The solid lines are the best fits of a double exponential function to the experimental data and the fitted parameters are summarized in Table 2. The bottom panels display the weighted residuals (W. residuals) from the fittings. The buffers contained 5 mM DDM and 200 mM KCl (*T* = 25 °C).

The proteolytic cleavage of KcsA C-terminal domain by chymotrypsin (amino acids 125–160) is known to destabilize the inner bundle gate, which adopts a looser structure in truncated form [9,59]. To test the sensitivity of the engineered double homo-FRET based sensor to the conformational dynamics of its inner gate, we compared the variation of <*r*>_TMR_ with pH between the full-length and truncated (Δ125) forms of TMR-labeled G116C W67 KcsA at 200 mM KCl. The enzymatic deletion of the 125–160 fragment of KcsA resulted in a higher <*r*>_TMR_ at pHs near neutrality, and in similar values when the channel is gradually open by the pH drop (Figure 7A). Since a decrease in the hydrodynamic volume of the protein is expected to reduce its steady-state anisotropy when there are no concomitant changes in the mean fluorescence lifetime of the fluorophore (as it was found experimentally to be the case here (Figure S8C)), it can be conclude that the small increase detected in <*r*>_TMR_ near neutrality must be due to a less efficient homo-FRET process among the conjugated dyes due to an increased flexibility of the C-terminal truncated protein. In this respect, it is also significant that the residual putative oblique J-dimer formation, which is critically dependent on the protein’s ability to keep a strict orientation of the covalently linked dyes, was also less favored in the truncated form compared to the full-length protein (Figure S9B), whereas H-type dimer formation at low pH was, in this case, essentially independent of the presence or absence of cytoplasmic CTD of KcsA (Figure S9A). On the other hand, the <*r*>_W67_ from the Trp 67 residue were identical for the full-length and truncated protein, indicating that there was no concomitant significant change on the pore-helix dynamics/average SF ion occupancy after the enzymatic cleavage at a saturating K^+^ concentration (Figure 7B).

The fluorescence depolarization kinetics of the full-length and truncated TMR-labeled G116C W67 KcsA proteins shed further light on their molecular rotation in solution. Representative anisotropy decay curves obtained at pH 3.5 and 7.0 are displayed in Figure 7C,D and a summary of the data analysis with a biexponential function is presented in Table 2.

Strikingly, the long rotational correlation time, φ_2_, of the truncated protein became pH-independent (φ_2_ = 42 ± 2 ns, *n* = 8) at variance with what is observed with the full-length protein, confirming the sensitivity of this time-resolved technique to the structural rearrangements undergone by the CTD of KcsA upon its gating at acidic pH: φ_2_(full-length)~1.4 × φ_2_(Δ125) at a pH of 3.5 whereas φ_2_(full-length)~2.4 × φ_2_(Δ125) at pH 7.0 (Table 2). These results are a strong indication that the full-length channel transitions from an overall very rigid and ellipsoid-like conformation at neutral pH to a more splayed, globular-like structure at a low pH in agreement with the literature [56,57]. It is also important to stress that a much faster initial depolarization of the emitted fluorescence was detected for the full-length compared to the truncated fluorescently-labeled protein at pH 7.0 (*r*(0)_full-length_ = 0.26 < *r*(0)_Δ__125_ = 0.30 at this pH (Table 2), confirming the stabilizing role of the C-terminal domain on the closed state of the channel at pH 7.0. This in turn, increases the efficiency of the intramolecular homo-FRET process due to a closer proximity between the TMR dyes attached at position 116 and ultimately leads to a lower <*r*>_TMR_ value (Figure 1C). Even though the conformational state (i.e., intersubunit distances) of the inner gate at pH 7.0 changes upon the deletion of the C-terminal domain, the network of amino acids involved in the pH sensing remains intact, and thus the activation p*K*_a_ values are almost identical between the full-length and Δ125 channels (Figure 7A). The C-terminal domain deletion weakens the van der Waals contacts (at the level of V115) at the 4-helix bundle crossing and therefore, the inner gate fails to efficiently close the channel [9]. Considering these results, it is reasonable to assume that the inner gate of the truncated protein adopts a looser conformation than the full-length version but is not completely open as in the pH 4.0 situation.

## 3. Discussion

In this work, we have engineered a double homo-FRET based sensor—TMR-labeled G116C W67 KcsA—to track the conformational dynamics related to pH-induced inner gating and its allosteric connection to the SF of this potassium channel. The measurements were performed with the detergent-solubilized protein, at low µM concentration and room temperature, thus complementing previous X-ray, EPR, and NMR studies but under milder experimental conditions. By combining the application of steady-state and time-resolved fluorescence anisotropy measurements with a careful control of the degree of protein labeling, we detected an efficient intramolecular homo-FRET process in the multiply TMR-labeled channels at the C116 position, which is responsive to two structural states (closed/open populations of the inner gate) that interconvert in solution in a pH-dependent manner. In contrast to hetero-FRET measurements, these assays are single-colored, i.e., they require labeling the tetrameric protein with a single extrinsic fluorophore, greatly simplifying sample preparation. In fact, one of the major obstacles preventing the wider application of hetero-FRET methodologies to study the conformational dynamics of oligomeric proteins has been the technical difficulties associated with position-specific fluorophore labeling in multimeric proteins [65]. The experimental readout of the performed homo-FRET studies, i.e., the steady-state fluorescence anisotropy of the chosen TMR reporter, is a concentration-independent ratiometric parameter that can be easily measured in a common spectrofluorometer or easily implemented in a plate reader, allowing the performance of high-throughput screening studies of ion channel gating.

By taking advantage of the sigmoidal dependence of the fluorescence anisotropy of the TMR dyes conjugated to the C116 position with pH, we were able to determine the activation p*K*_a_ of the TMR-labeled channels under different ionic conditions (Figure 6). A p*K*_a_ of ~4.2 was obtained in the presence of saturating K^+^ concentrations, in agreement with the proton dissociation constants described by both functional [15,66] and structural elucidation techniques [37,58,67] of KcsA. In contrast, the activation p*K*_a_ of the inner gate underwent an upward shift of almost 1 pH unit when the fluorescently labeled protein was studied in the presence of either 200 mM Rb^+^ or Na^+^. A similar effect was observed by Tilegenova and collaborators working with asolectin reconstituted channels and using electron paramagnetic resonance measurements [37], where they found that the activation p*K*_a_ was 4.3 in the presence of 150 mM KCl and 5.2 in the presence of 200 mM RbCl. This agreement reinforces our findings and highlights that the conformational dynamics of the inner gate of KcsA is not significantly perturbed by its reconstitution conditions (i.e., protein solubilized in detergent micelles versus embedded in a lipid bilayer).

**Figure 8:**
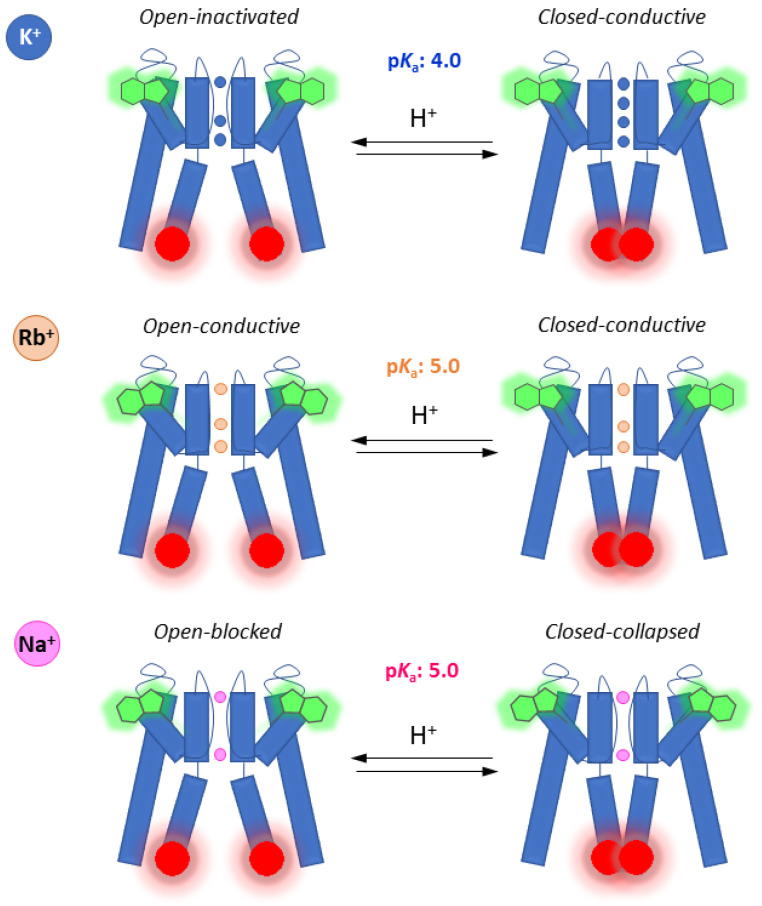
The ion occupancy at the S2 site of the SF contributes to controlling the allosteric crosstalk between the inner and outer gates of the KcsA potassium channel. Schematic representation of the allosteric communication between the inner and outer gates of the KcsA channel, reported by fluorescence anisotropy of the covalently bound TMR at position C116 (red) and the intrinsic fluorophore W67 (green), respectively. The conformation of the SF (outer gate) is sensitive to the different ion occupancy presented by K^+^, Rb^+^ and Na^+^. In the case of K^+^, its ability to bind with equal probability to the four binding sites at the SF (when the inner gate is in the closed conformation) leads to closer W67-W67 inter-subunit distances and an activation p*K*_a_ of ~4.0. On the other hand, the inner gate opened at lower [H^+^] (pK_a_ of ~5.0) when Rb^+^ or Na^+^ are bound to the SF at S1–S3–S4 or S1–S4 positions, respectively, i.e., when there was a loss of ion occupancy at the S2 site. The inability of both cations to interact with the S2 site at the outer gate is also characterized by higher W67-W67 inter-subunit distances.

The simultaneous presence of two independent reporters in the TMR-fluorescently labeled G116C W67 KcsA mutant provided a simple way to correlate the conformational changes undergone by its inner and outer gates under different experimental conditions, i.e., to study their allosteric coupling. The native W67 residue, strategically located at the pore helix and connected to the SF through a hydrogen bond interaction network, senses the conformation of the outer gate in terms of collapsed (200 mM NaCl) or conductive (200 mM KCl or RbCl) forms due to the presence of a highly efficient homo-FRET process among the four W67 residues (one in each subunit) [12]. Here, the <*r*>_W67_ values agreed with the expected values (~0.14–0.15 for a conductive SF; ~0.17–0.18 for a collapsed form) even when TMR was conjugated to the KcsA channels to different extents (Figure 6) thus confirming that the labeling of the protein did not affect *per se* the conformation adopted by the SF. Interestingly, although the SF of TMR-labeled G116C W67 KcsA shared a conductive conformation in the presence of a saturating concentration of K^+^ and Rb^+^, the activation p*K*_a_ of their inner gates differed by almost one pH unit. Taken all this information into account, it seems that the opening of the inner gate is hindered when the four binding sites of the SF are saturated with K^+^ and facilitated when the ion occupancy of the SF is lower (S1/S4 in Na^+^ or S1/S3/S4 in Rb^+^) (Figure 8). These results are consistent with previous EPR and electrophysiological studies that show a lower activation p*K*_a_ in the presence of K^+^, but not in Rb^+^ nor Cs^+^ [37,66], reinforcing the possible role of the S2 binding site occupancy in the allosteric communication between the inner and outer gates of KcsA.

The impact of the proteolytic removal of the C-terminal domain on the conformational dynamics of the inner gate of the TMR labeled G116C W67 KcsA mutant channel was also tested. EPR studies have suggested that the truncated channel is in the open state at pH 7.0 when solubilized in detergent micelles but closed when reconstituted in liposomes [68]. Here, the combination of the W67 and TMR information indicated that the interaction network defining the pH sensor is not greatly affected after the proteolytic removal of the CTD (i.e., the p*K*_a_ value obtained for the truncated protein remained almost identical to the full-length protein), even though the conformation of bundle-crossing of the TM2 helices at pH 7.0 is looser than in the full-length channel (higher <*r*>_TMR_ values). The differences between the truncated and full-length channel at neutral pH suggest the existence of a new intermediate state in the degree of opening of the intracellular mouth. This fact is in agreement with previous K^+^ binding assays of the detergent solubilized Δ125–160 KcsA channel, where the decrease on the affinity for the permeant cation at pH 7.0 was related to a change in the stability of the inner gate and its allosteric connection to the SF conformational dynamics [69].

Finally, the variation with pH of the absorption and fluorescence emission properties of TMR conjugated to G116C W67 KcsA channels at a high D:P provided two additional spectroscopic parameters (Abs_554_/Abs_520_ ratio and <λ>) that could be used to independently track its pH-induced gating. The propensity of rhodamine dyes, either in a free form [51,53] or conjugated to proteins [47,48], to form ground-state dimers with a variable geometry that are in equilibrium with the monomeric fluorophore has been extensively reported in the literature. The absorption spectra of rhodamines are known to be dependent on the concentration and the type of solvent used and therefore their spectral features inform about the geometrical arrangement of the dimer present in solution [70]. In our case, the formation of non-fluorescent H-type dimers (with parallel stacked chromophores) by the covalently linked TMR dyes was energetically favored at acidic pH (Figure 4A and Figure S6A), whereas residual putative oblique J-dimer formation (where the S_o_ → S_1_ transition dipole moments of the attached dyes are held on the same plane) could be detected near neutrality (Figure 4B and Figure S6B). This rich spectroscopic behavior, which was undisclosed in previous studies carried out with TMR-labeled KcsA [39,71,72], was found to be critically controlled by the conformational freedom of the CTD of these channels. This, in turn, is governed by the pH-switchable activation state (closed/open) of the inner gate and yielded similar activation p*K*_a_’s to the ones determined from using steady-state fluorescence anisotropy measurements, thus further corroborating our conclusions about the indirect influence of the ionic environment on the inner gate dynamics of KcsA through its effect on the degree of occupancy of site S2 of the SF.

In sum, our double-reporter system was able to unequivocally confirm the allosteric coupling between the inner and outer gates of KcsA. Furthermore, this homo-FRET based methodology provides a simple and accessible ratiometric fluorescent assay in the visible region which is ideally suited to address some of the persistent questions related to the regulation of the functional properties of the K^+^ channels.

## 4. Experimental Procedures

### 4.1. Heterologous Expression and Purification of KcsA

The pQE-60 and pQE-1 plasmids encoding the genes G116C W26/68/87/113F (G116C W67) and G116C wild-type (WT) *kcsa* and the pQE-30 plasmids encoding the genes W67 and WT *kcsa* were transformed in *E. coli* M15 (pRep4) strain. Then, purification of the channels was performed by immobilized metal affinity chromatography (IMAC) according to previously published protocols [12,73]. The purified proteins were kept refrigerated at 4 °C in 20 mM HEPES (Sigma-Aldrich, Madrid, Spain), 5 mM *N*-dodecy-β-d-maltoside (DDM, Merck, Madrid, Spain), 200 mM NaCl (Sigma-Aldrich, Madrid, Spain), pH 7.0 buffer until used. The protein concentration was determined spectrophotometrically at 280 nm using the following molar extinction coefficients: G116C W67 and W67 KcsA: 12,950 M^−1^ cm^−1^; WT and G116C KcsA: 34,950 M^−1^ cm^−1^ [74].

### 4.2. Functional and Structural Characterization of the Mutant Channels

For the functional characterization, inside-out patch clamp recordings were conducted on excised patches from asolectin (Sigma-Aldrich, Madrid, Spain) giant liposomes containing either WT, G116C WT, G116C W67, or TMR-labeled G116C W67 KcsA as reported previously [75], using an automated patch clamp system (Nanion Technologies, München, Germany) equipped with an external perfusion device, and afterwards analyzed with Clampfit 10.3 (Molecular Devices, Axon Instruments, San José, CA, USA).

The oligomeric status of the protein was checked by SDS polyacrylamide gel electrophoresis (SDS-PAGE, 13.5%) [76]. The stability of the purified proteins was tested by a thermal denaturation assay performed in a Varian Cary Eclipse spectrofluorometer (Agilent Technologies, Santa Clara, CA, USA) by recording the temperature dependence of the protein intrinsic emission fluorescence at 340 nm upon excitation at 280 nm, as previously described [77].

### 4.3. TMR Labeling of G116C W67 KcsA Mutant Protein

The labeling reaction of the G116C W67 channel with tetramethylrhodamine-5-maleimide (TRM-M, Invitrogen) was performed according to the manufacturer’s protocol (www.thermofisher.com, accessed on 20 Janurary 2020) usually at equimolar or 10-fold molar excess of TMR-M for 2 h at room temperature in 20 mM HEPES, 5 mM DDM, 200 mM NaCl buffer at pH 7.0. The excess of free dye was effectively removed by binding the conjugated His-tagged protein to the Ni^2+^-Sepharose resin followed by extensive washing and subsequent dialysis (20 mM HEPES, pH 7.0 with 5 mM DDM and 200 mM NaCl or 200 mM KCl) [12]. The efficiency of labeling (final dye-to-protein molar ratio, D:P) was determined by means of the absorption spectrum of the sample using a molar extinction coefficient of 83,871 M^−1^ cm^−1^ for TMR-labeled channels at 552 nm, whereas the protein concentration of the sample was quantified spectrophotometrically using the DC protein assay (Bio-Rad, Madrid, Spain). Typically, the final D:P of the TMR-labeled channels was approximately 0.12 and 0.45 for an equimolar and a 10-fold molar excess of dye used during the labeling reaction, respectively.

### 4.4. Preparation of the Samples

The influence of pH on the conjugated TMR and W67 fluorescence properties was studied in intervals of half pH unit by dialyzing the labeled protein samples (6 µM monomer based) in 10 mM succinic acid (Sigma-Aldrich, Madrid, Spain) buffers from pH 3.5 to pH 6.5 and 20 mM HEPES, pH 7.0 buffer. In all cases, the final pH was adjusted with *N*-methyl-d-glucamine. NaCl, RbCl, or KCl were added to the buffers according to the desired ionic condition to be tested, which was typically set at 200 mM final concentration unless otherwise indicated. The influence of the C-terminal domain on the results was tested by submitting a high D:P labeled stock of full-length channel (pH 7.0) to enzymatic cleavage with α-chymotrypsin-agarose beads (Sigma-Aldrich, Madrid, Spain) [69] and then dialyzing the truncated protein in the buffers mentioned above.

### 4.5. Steady-State and Time-Resolved Spectroscopic Characterization

The absorption spectra of the fluorescently labeled channels were obtained at room temperature using a single-beam spectrophotometer Agilent Cary 50 UV–Vis Spectrophotometer (Agilent Technologies, Santa Clara, CA, USA) and 1-cm pathlength quartz cuvettes. The spectra were corrected for the light scattering produced by the 5 mM DDM-containing buffers.

Steady-state fluorescence measurements were performed on a SLM-8000C spectrofluorometer (SLM–Aminco, Urbana, IL, USA) using 0.5-cm pathlength quartz cuvettes (Hellma, Müllheim, Germany). For TMR recordings, sample excitation was typically set at 490 nm. For the W67 characterization, the excitation wavelength was set at 295 nm for the emission spectra recordings and 300 nm for the anisotropy measurements. Typically, 4-nm and 8-nm slits were used in both the excitation and emission monochromators for spectra collection and steady-state fluorescence anisotropy measurements, respectively. Background intensities arising from the buffer with DDM were always subtracted.

The fluorescence spectral center-of-mass (intensity-weighted average emission wavelength, <λ>) of each emission spectrum was calculated according to,

(1) $$<\lambda >=\frac{\Sigma_{i}\lambda_{i}{Ι}_{i}}{\Sigma_{i}\lambda_{i}},$$

where ${Ι}_{i}$ is the fluorescence intensity of the sample measured at a wavelength λ_i_ [78]. The steady-state fluorescence anisotropy, <r>, of the fluorescently labeled KcsA mutants was calculated as

(2) $$<r>\;=\frac{I_{\mathrm{VV}\;}-G\cdot I_{\mathrm{VH}}}{I_{\mathrm{VV}\;}+2\;G\cdot I_{\mathrm{VH}}},$$

where *I*_VV_ and *I*_VH_ are the fluorescence intensities (blank subtracted) of the vertically and horizontally polarized emission, when the sample is excited with vertically polarized light, respectively, and the *G*-factor (*G* = *I*_HV_/*I*_HH_) is the instrument correction factor.

Time-resolved fluorescence measurements were performed according to the single photon-timing technique [79]. The TMR fluorescently labeled KcsA channels were excited at 488 nm using a BDS-SM-488FBE pulsed picosecond diode laser from Becker & Hickl (Berlin, Germany). The measurements were performed at 25 °C and the samples were kept under constant stirring. Data were analyzed using the TRFA Data Processer Advanced version 1.4 from the Scientific Software Technologies Centre (Belarusian State University, Minsk, Belarus). The fluorescence intensity and anisotropy decays were fitted with a sum of three and two exponentials, respectively, and the goodness of the fits were judged from obtaining a reduced χ^2^ < 1.3 and a random distribution of the weighted residuals and autocorrelation plots [22]. The intensity-weighted mean fluorescence lifetime, <τ> was calculated according to

(3) $$<\tau >\;=\frac{\sum_{i=1}^{n}\alpha_{i}\tau_{i}{}^{2}}{\sum_{i=1}^{n}\alpha_{i}\tau_{i}},$$

where $\alpha_{i}$ and $\tau_{i}$ are the normalized amplitude and the lifetime of the ith decay component, respectively.

### 4.6. Quantum Yield and Förster Radius Determination of the TMR Labeled KcsA Channel

The quantum yield of the TMR dye either free in solution or conjugated to a protein, was determined by the relative method using Rhodamine 101 as a reference (Q = 0.915 in ethanol) [80]. The Förster radius, *R*_0_, or the critical distance at which the transfer efficiency is 50% for an isolated donor–acceptor pair, was calculated for the TMR-labeled G116C W67 KcsA channel as in [12,22]. The quantum yield of the free TMR dye in methanol and in 5 mM DDM-containing buffer was 0.71 ± 0.01 (*n* = 3) and 0.40 ± 0.02 (*n* = 3), respectively. The quantum yield of the conjugated protein (D:P = 0.05)) in the DDM-containing detergent at pH = 7.0 was 0.21 ± 0.01 (*n* = 3). Assuming ε_max_(TMR) = 83,871 M^−1^ cm^−1^, the calculated Förster radius, *R*_o_, for a TMR-labeled G116C W67 KcsA channel was 44.1 Å at pH = 7.0 (the orientation factor was set to 2/3 and the refractive index of the medium was considered to be 1.4 for the DDM-containing buffer).

## Figures and Tables

**Table 1:** Influence of pH and degree of labeling (D:P) on the fluorescence anisotropy decay parameters for TMR-labeled G116C variant of W67 KcsA. The amplitudes, β*_i_* and rotational correlation times, φ*_i_*, were obtained by global fitting of a two-exponential function to the data, i.e., by linking φ_2_ between the anisotropy decay curves obtained for each pH at a low (D:P 0.12) and high degree of labeling (D:P 0.45) (λ_ex_ = 488 nm; λ_em_ = 575 nm). The buffers contained 5 mM DDM and 200 mM KCl (*T* = 25 °C).

pH	D:P	β_1_ *^a^*	φ_1_ *^b^* (ns)	β_2_ *^a^*	φ_2_ *^c^* (ns)	*r*(0) *^d^*	$\chi_{G}^{2}$
3.5	0.12	0.08	0.19	0.30	51 [49–55]	0.38	1.191
	0.45	0.09	0.23	0.25		0.34	
5.0	0.12	0.09	0.10	0.31	76 [71–84]	0.40	1.278
	0.45	0.06	0.22	0.21		0.27	
7.0	0.12	0.08	0.03	0.30	92 [85–103]	0.38	1.289
	0.45	0.06	0.23	0.20		0.26	

*^a^* β_1_, β_2_: ±0.01. *^b^* φ_1_: ±0.05 ns. *^c^* The square brackets indicate the 67% confidence interval of the global fitted long rotational correlation time, φ_2_. *^d^* The time-zero or initial anisotropy is the sum of the two amplitudes of the decay, *r*(0) = β_1_ + β_2_.

**Table 2:** Influence of the C-terminal truncation on the fluorescence anisotropy decay parameters for the detergent-solubilized TMR-labeled G116C variant of W67 KcsA. The amplitudes, β*_i_* and rotational correlation times, φ*_i_*, were obtained by fitting a two-exponential function to the anisotropy decay curves obtained for the C-terminal truncated (Δ125) or full-length (FL) fluorescently labeled protein (D:P = 0.60) at pH 3.5–6.5 (10 mM succinic acid, 5 mM DDM, 200 mM KCl) and pH 7.0 (20 mM Hepes, 5 mM DDM, 200 mM KCl) (*T* = 25 °C).

Protein	pH	β_1_ *^a^*	φ_1_ *^b^* (ns)	β_2_ *^a^*	φ_2_ *^c^* (ns)	*r*(0)	$\chi_{}^{2}$
Δ125	3.5	0.09	0.25	0.24	42 [40–44]	0.33	1.134
	4.0	0.08	0.30	0.23	43 [40–45]	0.31	1.134
	4.5	0.10	0.20	0.23	39 [37–42]	0.33	1.117
	5.0	0.09	0.23	0.21	41 [39–44]	0.30	1.153
	5.5	0.10	0.18	0.21	40 [38–42]	0.31	1.088
	6.0	0.09	0.23	0.22	39 [37–41]	0.31	1.110
	6.5	0.09	0.21	0.21	44 [41–46]	0.30	1.135
	7.0	0.09	0.20	0.21	45 [42–48]	0.30	1.184
FL	3.5	0.07	0.52	0.26	57 [53–61]	0.32	1.210
	7.0	0.07	0.16	0.18	99 [87–114]	0.26	1.033

*^a^* β_1_, β_2_: ±0.01. *^b^* φ_1_: ±0.05 ns. *^c^* The square brackets indicate the 67% confidence interval of the single fitted long rotational correlation time, φ_2_. *^d^* The time-zero or initial anisotropy is the sum of the two amplitudes of the decay, *r*(0) = β_1_ + β_2_.

## Data Availability

Data is contained in the article.

## Supplementary Materials

The following are available online at https://www.mdpi.com/article/10.3390/ijms222111954/s1.
