# Supplementary material for: Probing the Structural Dynamics of the Activation Gate of KcsA Using Homo-FRET Measurements
Source: Int J Mol Sci. 2021 Nov 4;22(21):11954. doi: 10.3390/ijms222111954 (PMC8584343; doi:10.3390/ijms222111954)
Supplement: Supplementary file 1 [file ijms-22-11954-s001.zip › ijms-1413290-supplementary.pdf]

Probing the Structural Dynamics of the Activation Gate of KcsA using homo-FRET Measurements

Supplementary information

Clara Díaz-García[1,2], Maria Lourdes Renart[3 *], José Antonio Poveda[3], Ana Marcela Giudici[3], José M. González-Ros[3], Manuel Prieto[1,2], Ana Coutinho[1,2,4 *].

[1] iBB, Institute for Bioengineering and Biosciences, Instituto Superior Técnico, Universidade de Lisboa, Lisboa, Portugal

[2] Associate laboratory i4HB – Institute for Health and Bioeconomy at Instituto Superior Técnico, Universidade de Lisboa, Lisboa, Portugal

[3] Instituto de Investigación, Desarrollo e Innovación en Biotecnología Sanitaria de Elche, Universidad Miguel Hernández, Elche, Alicante, Spain

[4] Dep. de Química e Bioquímica, Faculdade de Ciências, Universidade de Lisboa, Lisboa, Portugal

* To whom correspondence should be addressed. Emails: ana.coutinho@tecnico.ulisboa.pt and lrenart@umh.es.

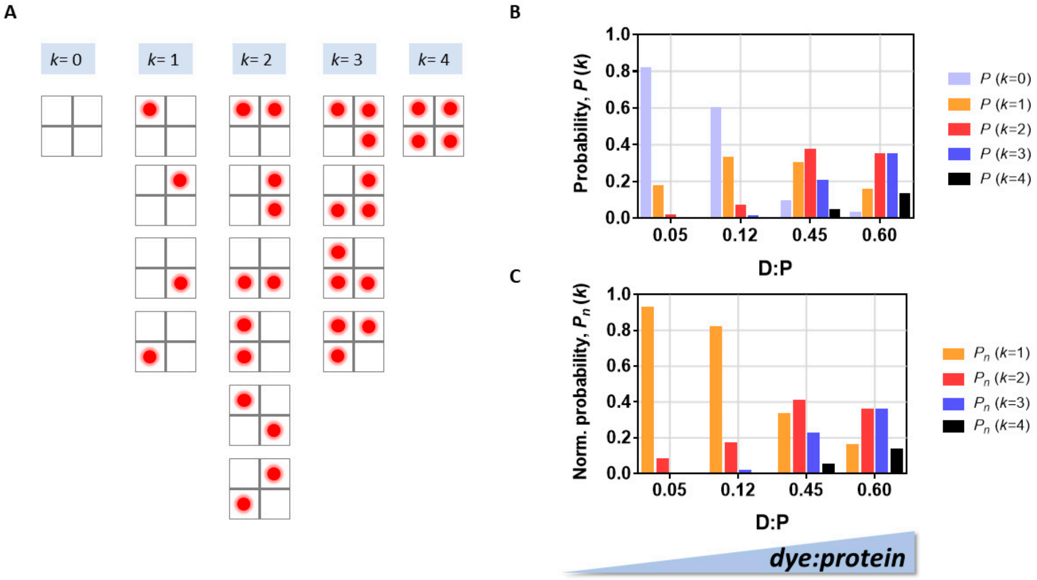

Figure S1. The average degree of labelling, D:P, of an oligomeric protein influences the final distribution of the fluorescently labeled species in solution. The random labeling of an oligomeric protein with one labeling site per subunit results in a heterogeneous population of fluorescently labeled species with a variable number of dyes, *k*, conjugated to each protein. **(A)** Considering a homo-tetramer, the probability distribution of unlabeled proteins *P* (*k*= 0), single-labelled proteins *P* (*k*= 1), double-labelled proteins *P* (*k*= 2), triple-labelled proteins *P* (*k*=3) and fully labelled proteins *P* (*k*=4) is described by the binomial theorem. **(B)** Upon increasing the final D:P molar ratio, the dominant species progressively shift from being the unlabeled proteins to a more complex solution enriched in multiply labeled species. **(C)** Since the unlabeled oligomers are non-fluorescent, the probability distribution needs to be re-normalized by eliminating their contribution to the total population of protein species present in solution.

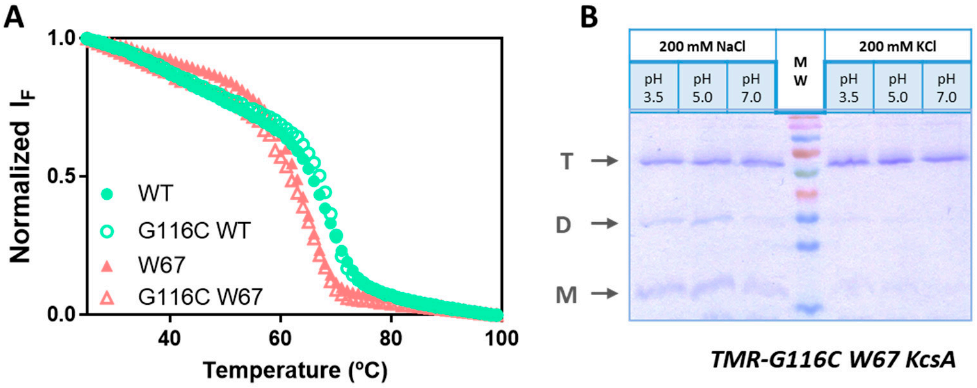

Figure S2. The mutation G116C minimally perturbs the structural properties of the wild-type (WT) and W67 KcsA channels. (**A**) Protein thermal stability assays show that the quadruple mutations W26,68,87,113F only slightly decrease the thermal stability of W67 and G116C W67 KcsA channels (t_m= 64 °C) compared to the WT and G116C WT proteins (t_m= 69 °C). The changes in the protein´s intrinsic fluorescence emission with the temperature was monitored at 340 nm (λ_{ex} = 280 nm). The samples contained 1 µM protein solubilized in 20 mM HEPES, pH 7 buffer with 5 mM DDM and 100 mM NaCl. (**B**) SDS-PAGE (13.5%) analysis of the tetrameric integrity of TMR-labelled G116C W67 KcsA channels (D:P= 0.45) at variable pH and in the presence of either 200 NaCl or KCl. The tetramer (T) is the predominant band in the gel, and only a residual presence of dimers (D) and monomers (M) is observed at low pH and in the presence of 200 mM NaCl.

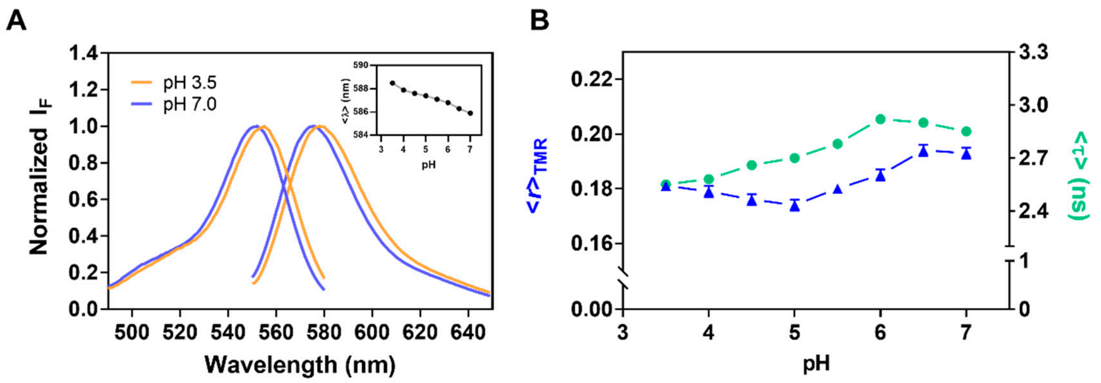

Figure S3. Influence of pH on the photophysical properties of the free TMR-M dye. (A) Normalized excitation (λ_{em}= 590 nm) and emission (λ_{ex}= 490 nm) spectra for the free TMR-M dye in detergent micelles at pH 3.5 and 7.0. The buffers contained 5mM DDM and 100 mM NaCl (*T*= 25 °C). The inset represents the variation of the center-of-mass from the emission spectra, <λ>, along the pH titration curve. Both the excitation and emission spectra of the free dye underwent a 2 – 3 nm red-shift upon acidification of the media from pH 7.0 to 3.5. **(B)** Both the steady-state fluorescence anisotropy, $<r>_{TMR}$, and intensity-weighted mean fluorescence lifetime, <τ>, of the free dye respond modestly to the protonation of its carboxyl group as the pH decreases.

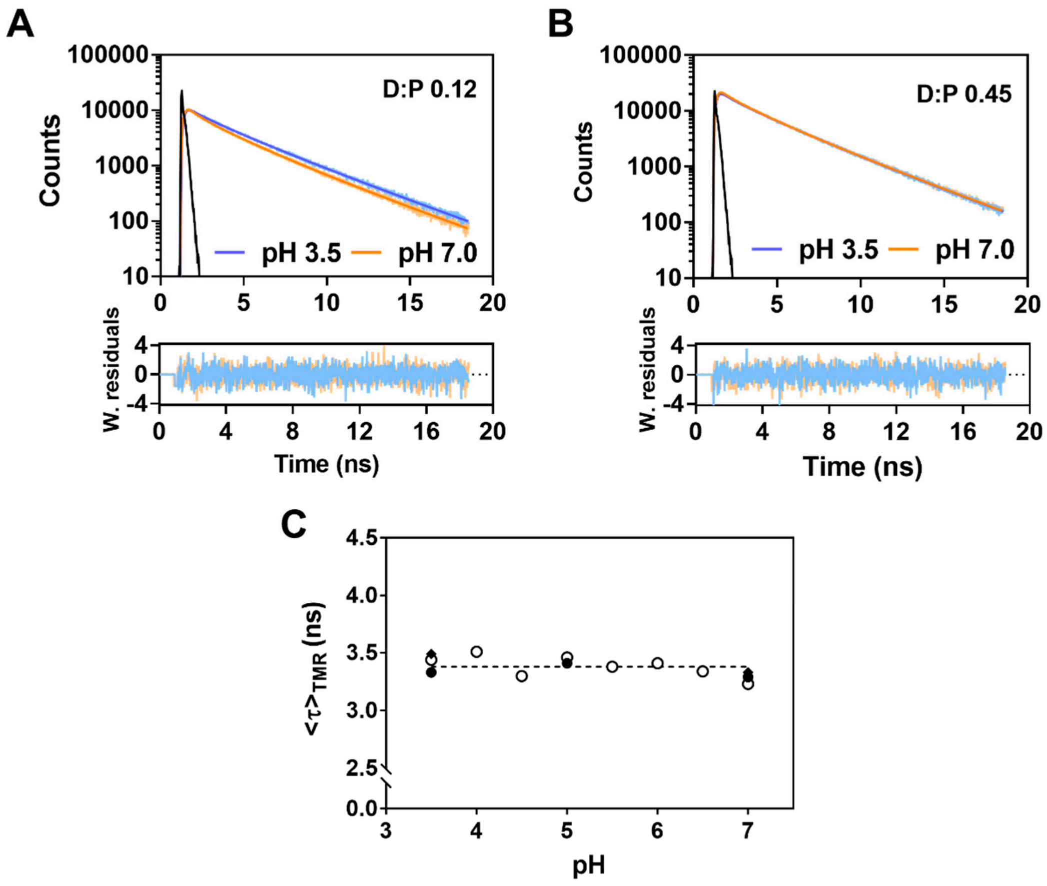

Figure S4. Influence of pH and degree of labelling, D:P, on the fluorescence intensity decays of TMR-labelled G116C W67 KcsA channels. Representative time-resolved fluorescence intensity decays (λ_{ex}= 488 nm; λ_{em}= 575 nm) for 6 µM TMR-labelled channels at pH 3.5 and 7.0 for a **(A)** D:P 0.12 and **(B)** D:P 0.45**.** The solid lines are the best fits of a three-exponential curve to the experimental data; the weighted residuals (W. residuals) are randomly distributed around zero showing the goodness of the fits. The instrument response function, IRF, are displayed as black curves. **(C)** The intensity-weighted mean fluorescence lifetime (Eq. 3) of TMR conjugated to G116C W67 KcsA protein, $<\tau>_{TMR}$, prepared with variable degrees of labelling (D:P 0.05 (◆), 0.12 (○) or 0.45 (●)) is independent of pH. The dashed line ($<\tau>$= 3.4 ± 0.1 ns) is just a guide to the eye. The buffers contained 5 mM DDM and 200 mM KCl (*T*= 25 ºC).

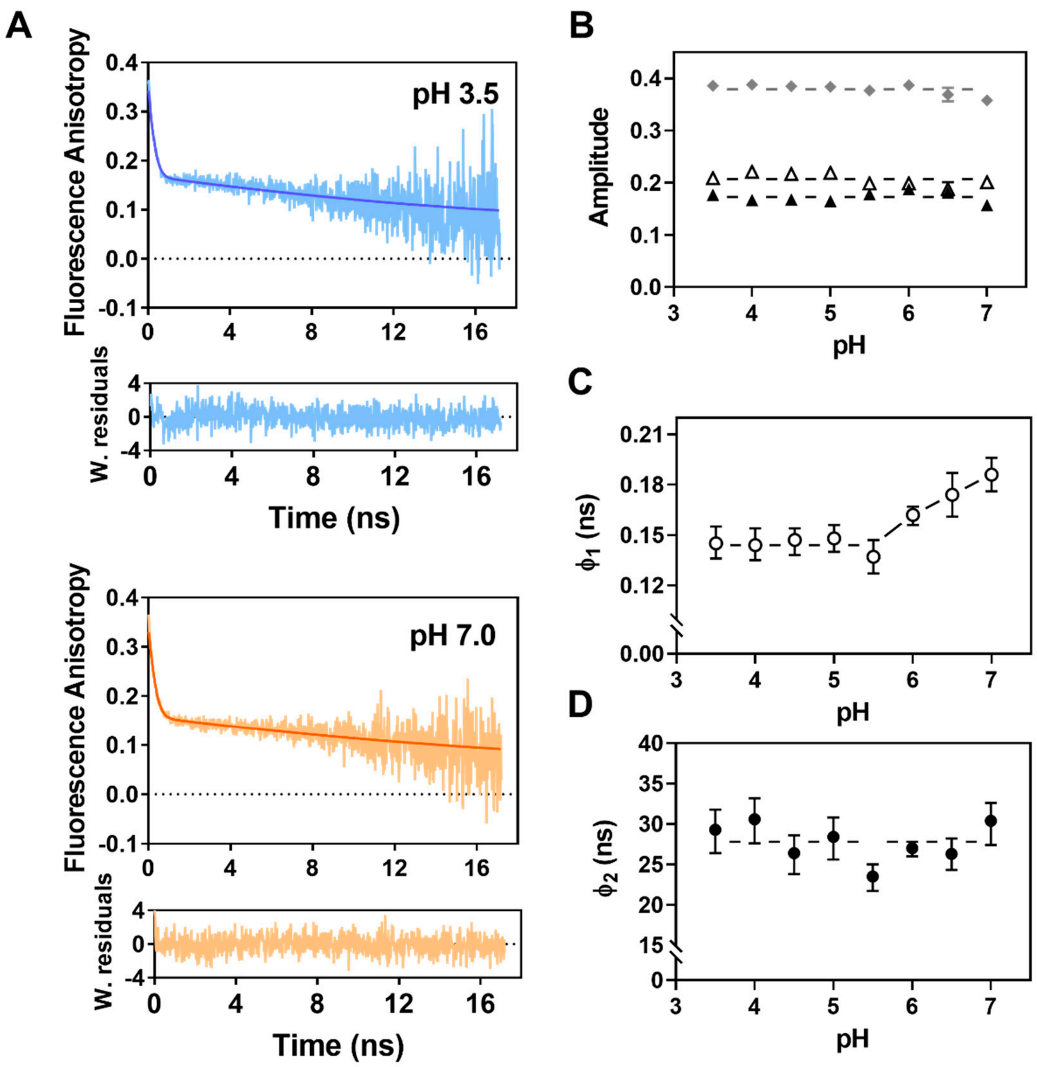

Figure S5. Influence of pH on the rotational dynamics of the detergent solubilized free TMR-M dye. (A) Representative time-resolved fluorescence anisotropy decays (λ_{ex}= 488 nm; λ_{em}= 575 nm) for 0.5 µM free TMR-M dye at pH 3.5 and 7.0**.** The buffers contained 5 mM DDM and 100 mM NaCl (*T*= 25 °C). The solid lines are the best fits of a two-exponential curve to the experimental data. **(B)** In all cases, the time-zero anisotropy, $r(0)= \beta_1 + \beta_2= 0.379 \pm 0.001$ (n= 8) is very close to the fundamental anisotropy of the dye ($r_o \approx 0.4$ [1]). The amplitudes β_1 and β_2 are represented by △ and ▲, respectively and *r*(0) is represented with ◆. **(C)** The fast rotational correlation time, (ϕ_1, represented by ○) in the 140 – 190 ps range, was assigned to the local rotational dynamics of the detergent-solubilized free dye itself. The small increase of ϕ_1 with pH is probably related to the ionization of the carboxyl group of TMR-M, allowing for the formation of an ionic pair with the ammonium group of the zwitterionic form of the dye which, in turn, slows down the depolarization of the emitted fluorescence by rotation of the xanthene group at neutral pH. **(D)** The slow overall tumbling of the DDM micelles in solution ((ϕ_2, represented by ●), with an average long rotational correlation time of ϕ_2= 27.8 ± 2.4 ns (dashed line), was found to be independent of pH. The error bars of the rotational correlation times correspond to the 67% confidence intervals of the fitted parameters. It

should be noted that the lifetime, τ, of the fluorescent probe plays an important role in the measured correlation times. In fact, as a rule-of-thumb, the approximate range of recoverable rotational correlation times with high precision lies within $0.1\ \tau < \phi < 10\ \tau$ [2]. Due to the large molecular weight of the DDM micelles in solution (~ 50 kDa [3]), their rotational correlation time is expected to be much longer than the mean fluorescence lifetime of the detergent-solubilized free TMR-M dye (Figure S2B), precluding its determination with a low uncertainty.

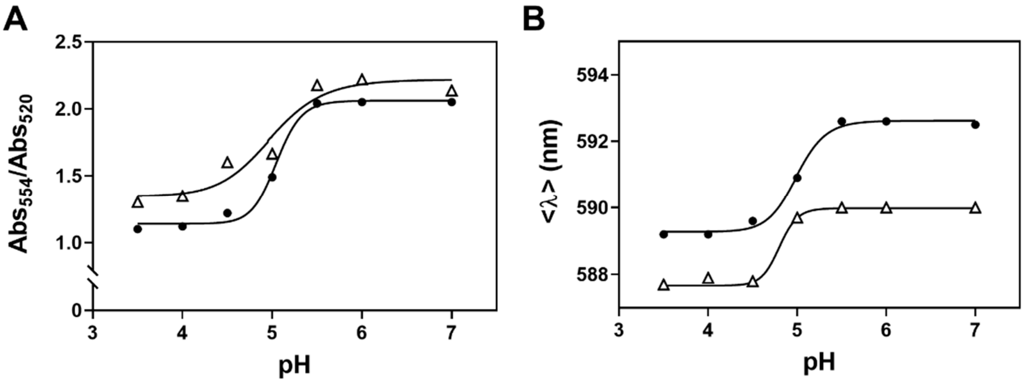

Figure S6. Influence of ionic environment on the variation of the spectroscopic properties of TMR-labelled G116C W67 KcsA with pH. (A) The extent of H-type ground-state dimer formation by the conjugated TMR probes was evaluated from the Abs_{554}/Abs_{520} ratio as a function of pH. **(B)** The residual self-association of the TMR conjugated probes into putative fluorescent oblique J-dimers was evaluated from its spectral center-or-mass, <λ>, variation with pH. The measurements were performed in the presence of 200 mM RbCl (D:P 0.38, △) and 200 mM NaCl (D:P 0.45, ●) (*T*= 25 °C). The solid lines correspond to the best fits of a Hill equation to the sigmoidal behavior of each data set with pH, giving a (A) $pK_a(Rb^+)$= 5.0 ± 0.2 and $pK_a(Na^+)$= 5.06 ± 0.04, and (B) $pK_a(Rb^+)$= 4.8 ± 0.05, $pK_a(Na^+)$= 5.00 ± 0.04, respectively.

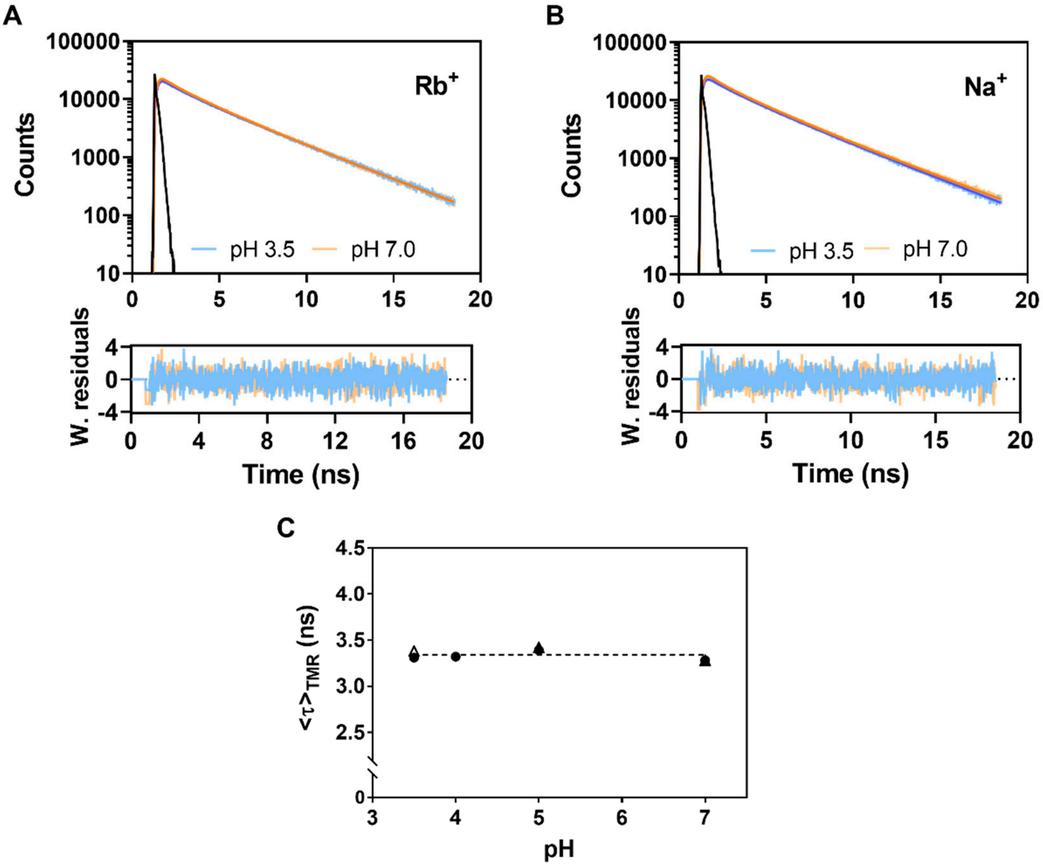

Figure S7. Influence of ionic environment and pH on the fluorescence intensity decays of TMR-labelled G116C W67 KcsA channels. Representative time-resolved fluorescence intensity decays (λ_{ex}= 488 nm; λ_{em}= 575 nm) for TMR-labelled channels in the presence of 200 mM **(A)** RbCl (D:P 0.38) and **(B)** NaCl (D:P 0.45) at pH 3.5 and 7.0 (T= 25 °C). The solid lines are the best fits of a three-exponential curve to the experimental data. The instrument response functions, IRF, are displayed as black curves. **(C)** The pH and ionic composition of the medium does not affect the intensity-weighted mean fluorescence lifetime, $<\tau>_{TMR}$, of fluorescently labelled G116C W67 KcsA in 200 mM RbCl (△) or NaCl (●). The dashed line ($<\tau>$= 3.3 $\pm$ 0.1 ns) is just a guide to the eye.

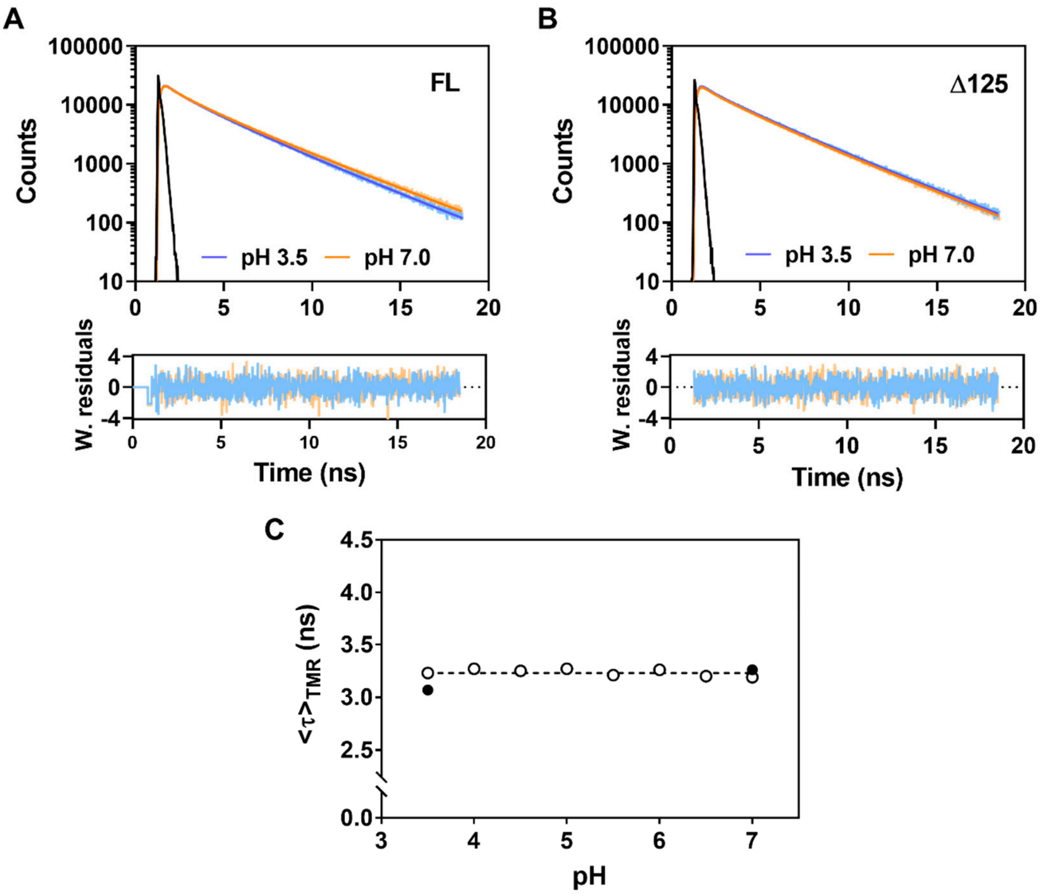

Figure S8. Influence of C-terminal truncation on the fluorescence intensity decays of TMR-labelled G116C W67 KcsA channels. Representative time-resolved fluorescence intensity decays (λ_{ex}= 488nm; λ_{em}= 575nm) for **(A)** full-length (FL) and **(B)** C-terminally truncated (Δ125) TMR-labelled channels (D:P= 0.60) at pH 3.5 and 7.0. The solid lines are the best fits of a three-exponential curve to the experimental data. The instrument response functions, IRF, are displayed as black curves. **(C)** The pH does not affect the intensity-weighted mean fluorescence lifetime, $<\tau>_{TMR}$, of the FL (●) and truncated fluorescently labelled G116C W67 KcsA protein (○). The dashed line ($<\tau>$= 3.2 ± 0.1 ns) is just a guide to the eye. The buffer contained 5mM DDM and 200mM KCl (*T*= 25 °C).

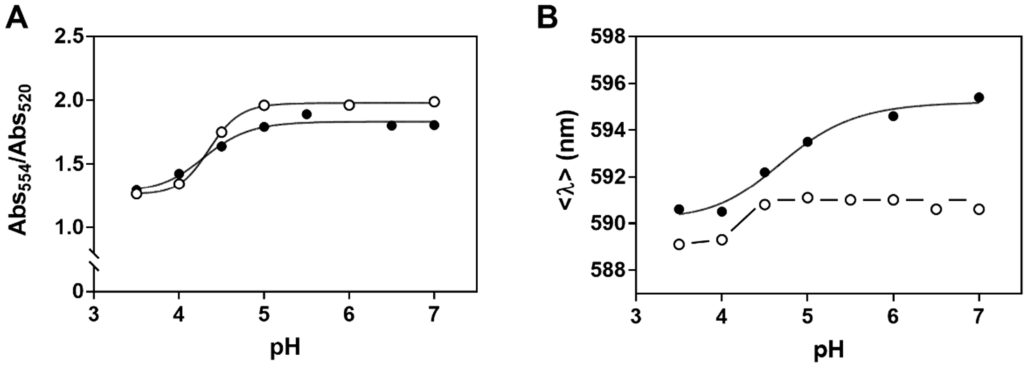

Figure S9. Influence of C-terminal truncation on the variation of the spectroscopic properties of TMR-labelled G116C W67 KcsA with pH. The measurements were carried out with full-length G116C W67 proteins conjugated with TMR (D:P 0.60) (FL, ●) and after its C-terminal truncation by proteolytic treatment with chymotrypsin (Δ125, ○) at variable pH and in the presence of 200 mM KCl (*T*= 25 °C). **(A)** The CTD of the channels does not impact the extent of H-type ground-state dimer formation by the conjugated TMR probes at low pH as evaluated from the Abs_{554}/Abs_{520} ratio. The solid lines correspond to the best fits of a Hill equation to the sigmoidal behavior of the absorbance ratio with pH with pK_a(FL)= 4.3 ± 0.1 and pK_a(Δ125)= 4.34 ± 0.03, respectively. **(B)** At variance, the residual self-association of the TMR conjugated probes into putative fluorescent oblique J-dimers, revealed by the red-shift detected on their emission spectra at neutral pHs, is much more pronounced for the FL compared to the truncated protein (the <λ> measurements of the Δ125 protein are not fitted due to the slight variation of the parameter). This result suggests that the later (Δ125) displays a larger conformational flexibility at position Cys116 than the former (FL) because the conjugated dyes must be held at specific orientations in the closed conformation of the channel for possible J-dimer-like dimerization.

References

1. Jameson, D.M.; Ross, J.A. Fluorescence Polarization/Anisotropy in Diagnostics and Imaging. *Chem. Rev.* **2010**, *110*, 2685–2708.

2. Wahl, P. Analysis of fluorescence anisotropy decays by a least square method. *Biophys. Chem.* **1979**, *10*, 91–104.

3. Oliver, R.C.; Lipfert, J.; Fox, D.A.; Lo, R.H.; Doniach, S.; Columbus, L. Dependence of Micelle Size and Shape on Detergent Alkyl Chain Length and Head Group. *PLoS One.* **2013**, *8*, e62488.
